# Effect of Cocaine on HIV Infection and Inflammasome Gene Expression Profile in HIV Infected Macrophages

**DOI:** 10.1038/srep27864

**Published:** 2016-06-20

**Authors:** Venkata Subba Rao Atluri, Sudheesh Pilakka-Kanthikeel, Gabriella Garcia, Rahul Dev Jayant, Vidya Sagar, Thangavel Samikkannu, Adriana Yndart, Madhavan Nair

**Affiliations:** 1 Department of Immunology, Institute of NeuroImmune Pharmacology, Herbert Wertheim College of Medicine, Florida International University, Miami, FL-33199, USA

## Abstract

We have observed significantly increased HIV infection in HIV infected macrophages in the presence of cocaine that could be due to the downregulation of BST2 restriction factor in these cells. In human inflammasome PCR array, among different involved in inflammasome formation, in HIV infected macrophages in the presence of cocaine, we have observed significant upregulation of NLRP3, AIM2 genes and downstream genes IL-1β and PTGS2. Whereas negative regulatory gene MEFV was upregulated, CD40LG and PYDC1 were significantly downregulated. Among various NOD like receptors, NOD2 was significantly upregulated in both HIV alone and HIV plus cocaine treated cells. In the downstream genes, chemokine (C-C motif) ligand 2 (CCL2), CCL7 and IL-6 were significantly up regulated in HIV plus cocaine treated macrophages. We have also observed significant ROS production (in HIV and/or cocaine treated cells) which is one of the indirect-activators of inflammasomes formation. Further, we have observed early apoptosis in HIV alone and HIV plus cocaine treated macrophages which may be resultant of inflammasome formation and cspase-1 activation. These results indicate that in case of HIV infected macrophages exposed to cocaine, increased ROS production and IL-1β transcription serve as an activators for the formation of NLRP3 and AIM2 mediated inflammasomes that leads to caspase 1 mediated apoptosis.

Human immunodeficiency virus (HIV) enters the brain shortly after the infection through the infected peripheral blood monocytes/macrophages traversing across the blood brain barrier (BBB) resulting in infection of microglia, and to lesser extent astrocytes in the brain which is a hallmark of acquired immunodeficiency syndrome (AIDS)-related neuroinflammation. These cells produce cytokines, chemokines and viral proteins leading to the neuroinflammation, and eventual neuronal damage^1^,^2^. The neuronal damage is further exacerbated in the presence of drugs of abuse such as cocaine^3^,^4^. Understanding the inflammasome activators, pattern and other pathogenic mechanisms in HIV infected macrophages in the presence of cocaine will further help in understanding the neuropathogenic mechanisms of HIV infection in cocaine abusers.

Innate immunity is the first line of defense discriminating host proteins and foreign bodies (microorganisms), by sensing the signals of “danger”, such as pathogens (pathogen-associated molecular patterns [PAMPs]) or host-derived signals of cellular stress. The innate immune system detects viral infection/danger signals largely through germline-encoded pattern recognition receptors (PRRs) present either on cell surface or within the intracellular compartment. The PRRs include Toll-like receptors (TLRs), the retinoic acid-inducible gene 1 like receptors (RLRs), the nucleotide oligomerization domain-like receptors (NLRs, also called NACHT, LRR and PYD domain proteins) and cytosolic DNA sensors. In few instances, while viral proteins are the triggers of these receptors, viral nucleic acids are the predominant activators. In response to extracellular signals, TLRs induce a signaling cascade that leads to cellular activation and production of inflammatory cytokines [Tumor necrosis factor (TNF), Interleukin (IL)-6, IL-8 and type 1 interferons]^5^. In response to danger signals produced into the host cell cytosol, Nod-like receptors (NLRs) come into the action and NLR nucleotide-binding oligomerization domain containing protein 1 and 2 (NOD1 and 2) induce a signaling cascade, similar to TLRs and results in inflammatory cytokine production^6^. Recognition of host or microorganism danger signals trigger the formation of a multiprotein complex called the inflammasome, that contains caspase 1^7^,^8^ (Fig. 1). Inflammasome oligomerization requires two signals, a priming signal that results in the transcription of interleukin-1 beta (IL-1β) and Interleukin 18 (IL-18), and a second signal that promotes indirect activation of the inflammasome such as reactive oxygen species (ROS), ion or membrane perturbations, or extracellular adenosine triphosphate (ATP).

In response to various activators, Apoptosis-associated speck-like protein containing a CARD (PYCARD), absent in melanoma 2 (AIM2) and members of the NLR family [NLR Family, Pyrin Domain Containing 1 (NLRP1), NLRP3, and NLR family CARD domain-containing protein 4 (NLRC4)] can self-oligomerize via homotypic NACHT domain interactions to high-molecular weight complexes (probably hexamers or heptamers), that trigger caspase-1 (inflammatory caspases) auto-activation^9^,^10^. Therefore these NLR multimolecular complexes are termed “inflammasomes,” as they control inflammatory caspases. Activated caspase-1 controls the maturation and secretion of proinflammatory cytokines such as IL-1β and IL-18, which direct host responses to infection and injury. IL-1β participates in the generation of systemic and local responses to infection and injury by generating fever, activating lymphocytes and by promoting leukocyte infiltration at sites of infection or injury. Whereas, IL-18 induces IFN-γ production and contributes to T-helper 1 (Th1) cell polarization.The exact composition of an inflammasome depends on the activator (various microbial and endogenous stimuli) which initiates inflammasome assembly. So far, four inflammasomes have been identified based on the NLR protein that they contain - the NLRP1 (NALP1) inflammasome, the NLRP3 (NALP3) inflammasome, the IPAF (NLRC4) inflammasome and the recently identified AIM2 inflammasome (does not contain any NLR protein).

HIV infection of macrophages sets up inflammation at the cell level but through unexpected mechanisms and also, cocaine interact to increase the HIV infection levels in macrophages. Therefore, understanding the effect of cocaine on HIV infection, inflammasomes and regulated genes expression in HIV infected macrophages may provide insights into the development of potential therapeutic targets for HIV-1 associated neurocognitive disorders in cocaine abusers. To understand the increased HIV infection levels in cocaine exposed macrophages, we have analyzed the expression of HIV restriction factors [apolipoprotein B mRNA-editing enzyme catalytic polypeptide-like 3G (APOBEC3G), Bone Marrow Stromal Cell Antigen 2 (BST2) and Tripartite motif-containing protein 5 (TRIM5) alpha] in these cells. As production of ROS is one of the activators of inflammasomes formation, we have also investigated the production of ROS in these macrophages. Further, we have also investigated the apoptosis of HIV infected macrophages in combination with cocaine which could be due to the activation of these inflammasomes.

## Materials and Methods

### Isolation and culture of peripheral blood mononuclear cells (PBMC)

PBMC were isolated from the leukopacks (buffy coats) which were commercially obtained from the community blood bank (One Blood, Miami, FL, USA). The buffy coat was diluted with phosphate buffer saline (PBS) (Invitrogen, Gaithersburg, MD) at room temperature. The diluted buffy coat was overlaid on top of the Ficoll-Histopaque without disturbing the interface of the two liquids. Samples were centrifuged at 1,200 g for 20 min. at room temperature with break off settings in the centrifuge (acceleration = 1, deceleration = 0). The PBMC layer at the interface was collected and cells were washed once with PBS. The pellet was resuspended in ACK lysing buffer for the lysis of red blood cells in samples and incubated on ice for 15 min. Cells were washed with PBS and total cell number and cell viability were determined by trypan blue exclusion (Sigma, St. Louis, MO) in a hemocytometer counting chamber. PBMC were re-suspended in culture medium containing Roswell Park Memorial Institute (RPMI) 1640 (Life Technologies, Gaithersburg, MD), 25 mM 4-(2-hydroxyethyl)-1-piperazineethanesulfonic acid (HEPES) (Sigma, St. Louis, MO), 2 mM glutamine (Sigma, St. Louis, MO), 100 μg streptomycin (Sigma, St. Louis, MO), 100 U penicillin (Sigma, St. Louis, MO), and 10% fetal bovine serum (Life Technologies, Gaithersburg, MD).

### Differentiation of monocytes into monocyte derived macrophages (MDMs)

After 2 h of incubation, non-adherent cells were removed and the T75 flasks containing adherent monocytes were washed with PBS twice and cultured in flasks for 7–10 days in the presence of macrophage colony stimulation factor (MCSF) (2 ng/ml; Sigma) at a density of 10^6^ cells/ml. Cells were 98% macrophages as judged by morphology.

### Monocyte derived macrophages infection with HIV-1 in the presence of cocaine

These adherent macrophages were transferred into 6-well culture plates (1 × 10^6^ cells/well) and infected with different amounts (0.5–2 MOI) of HIV-1Ba-L (clade B) (NIH AIDS Reagent Program Cat. # 510) for overnight at 37 °C using previously established protocols with few modifications^11^,^12^. After infection, unabsorbed virus was washed away using PBS, and cells were cultured for 10 days in the presence or absence of cocaine (1–2 μM) along with MCSF. HIV at 0.5 MOI and cocaine at 1 μM concentration were determined to use for all the experiments based on our dose response study for cytotoxicity and HIV infectivity.

### MTT cell viability assay

The MTT cell viability assay was carried out to confirm the amount of HIV and/or cocaine we have used is not toxic as described by Rao *et al*. with few modifications^13^. After 10 days of HIV infection and/or treatment with different concentrations of cocaine (1 μM and 2μM), 6-well plates were given media change with one ml medium and 100 μl of 3-(4,5-Dimethylthiazol-2-Yl)-2,5-Diphenyltetrazolium Bromide (MTT) (100 mg MTT/20 ml PBS) was added for each well and incubated at 37 °C for 3 hours. After that, one volume of stop mix solution was added and rocked for about 2 hours, centrifuged and the optical density of the solubilized formazan was determined spectrophotometrically by measuring the absorbance at 550 nm after transferring into the 96 well plate. The optical density of formazan in each well is directly proportional to the cell viability and utilized for calculations.

### Measurement of HIV infectivity in macrophages in the presence of cocaine

After 10 days of HIV infection and/or cocaine treatment, macrophages were harvested and the supernatant was used to measure the p24 production using the p24 antigen ELISA kit (ZeptoMetrix Corp. Cat # 0801200). The manufacturer’s kit protocol was followed to detect p24 antigen in the HIV infected and HIV plus cocaine treated macrophages.

### Western blot assay for HIV restriction factors protein expression

To investigate the mechanism behind the increased HIV infection levels observed in macrophages exposed to the cocaine, we have quantified the protein expression of different HIV restriction factors in these cells. Protein was isolated from the cell pellets of the macrophages treated with HIV and/or cocaine. For SDS-PAGE, similar amounts of control and test group cellular protein, typically 40 μg per lane were used. All the protein samples were separated by using Any KD Mini-Protean TGX precast Gels (Bio-Rad, Cat # 456–9034). Proteins were transferred to nitrocellulose membranes and membranes were blocked with 5% skimmed milk in TBS-T (20 mM Tris buffer, pH 7.5, 0.5 M NaCl, 0.1% Tween 20) for 1 hr. at room temperature and incubated at 4 °C overnight in the primary antibody diluted in 2% skimmed milk in TBS-T. The primary antibodies used were as follows: anti-APOBEC3G antibody (Millipore cat # ABE607, 1/5000), anti-TRIM5 alpha antibody (Millipore cat # ABF206, 1/5000), anti- BST2 (bone marrow stromal cell antigen 2) antibody (Millipore cat # ABC149, 1/5000). Subsequently, blots were washed in TBS-T (4 times, 10 min each) and incubated for 1 hr at room temperature in horseradish peroxidase-goat anti-rabbit antibody (Promega, Cat # W401B) diluted 1/2500 in 2% skim milk in TBS-T. After additional washings, protein bands were detected by chemiluminiscence using SuperSignal West Pico Luminol/Enhancer (Thermo Scientific, Cat # 1856136) and SuperSignal West Pico substrate (Thermo Scientific, Cat # 1856135). Band density was measured by using ImageJ software.

### mRNA extraction and first strand cDNA synthesis

After 10 days of HIV infection and/or co-treatment with optimum concentration of cocaine as described above, macrophages were harvested and the pellet was used for the mRNA isolation using illustra triplePrep Kit (GE Healthcare Life Sciences, UK; Cat # 28- 9425-44) and on-column DNase treatment step was also performed in the procedure. Purity and concentration of the RNA was measured by microspot RNA reader (Synergy HT Multi-Mode Microplate Reader from BioTek, US) and RNAs with an OD260 nm/OD280 nm absorbance ratio of at least 2.0 were used for PCR array. One microgram of RNA from all the control and test groups (cocaine treated, HIV infected and HIV infected in the presence of cocaine) was used for the first strand cDNA synthesis using SABiosciences’s RT2 First Strand Kit (Cat. # 330401) as described earlier^14^,^15^. Genomic DNA elimination step was performed prior to reverse transcription.

### Inflammasome PCR array

Monocyte derived macrophages prepared as above were grown in 6 well culture plates (1 × 10^6^ cells/well). These cells were infected with optimized concentration (0.5 MOI) of HIV-1Ba-L for overnight at 37 °C. Unabsorbed virus was washed away using PBS, and cells were cultured for 10 days in the presence or absence of optimized concentration of cocaine (1 μM) along with MCSF. mRNA was extracted and first strand cDNA was synthesized as described above and these cDNA were used for the inflammasome gene expression profile. Inflammasomes profiling in HIV infected and/or cocaine treated macrophages was done using 96 well format Human Inflammasomes RT^2^ Profiler™ PCR Array Kit (SABiosciences, Cat # PAHS-097ZA) using Stratagene Mx3000p qRT-PCR instrument. The Human Inflammasomes PCR array interrogates 84 genes involved in the function of inflammasomes, protein complexes involved in innate immunity, as well as general NOD-like receptor (NLR) signaling. This kit was chosen because it includes diverse genes encoding inflammasome components as well as genes involved in downstream signaling and inhibition of inflammasome function. In addition, this array includes other NLR family members, which may potentially form additional inflammasomes, and their downstream signaling genes. Table 1 shows the list of inflammasomes, NOD-like receptors, pro-inflammatory caspase genes and negative regulating and down-stream regulating genes we have investigated. Relative abundance of each mRNA species was assessed using RT2 SYBR Green/ROX PCR Master mix (SABiosciences, Cat # 330520) and aliquoted in equal volumes (25 μl) to each well of the real-time PCR arrays. The real-time PCR cycling program (as indicated by the manufacturer) was run on a Stratagene Mx3000p qRT-PCR thermal cycler. The threshold cycle (Ct) of each gene was determined by using the Stratagene MaxPro software. The threshold and baseline were set manually according to the manufacturer’s instructions. Ct data were uploaded into the data analysis template on the manufacturer’s website. The relative expression of each gene in HIV and/or cocaine treated macrophages was calculated using ΔΔCT method with five housekeeping genes and compared with the expression in control cells. Controls are also included on each array for genomic DNA contamination, RNA quality, and general PCR performance.

### Effect of HIV-1 and/or cocaine on ROS production in macrophages

Reactive oxygen species (ROS) production is known as one of the mechanisms responsible for the activation of inflammasomes. Therefore, we hypothesize that exposure of HIV infected macrophages to the cocaine increases ROS production that may contribute for the upregulation of inflammasome forming genes expression. ROS production following exposure to HIV-1 and/or cocaine in macrophages were detected using dichloro fluorescein diacetate assay (DCF-DA; Molecular Probes, Eugene, OR, USA) using the established protocol^16^ with few modifications. Cells were cultured in 6-well plates (10^6^ cells/well) overnight to allow them to adhere on the surface of the well. The next day, cells were treated with optimized concentration of HIV-1 and/or cocaine for 10 days. After 10 days of infection/treatment, cells (100,000 cells/well) were transferred into the 96 well plates along with its media and incubated overnight. Next day, negative control cells were treated with antioxidant, catalase (0.001 mg) for 2 h. Next, cells were treated with DCF-DA (100 mM) for 1 h at 37 °C and finally read in a BioTek Synergy HT microplate reader (excitation 485 nm and emission 528 nm; BioTek, Winooski, VT, USA). Cells treated with H_2_O_2_ for 2 h was considered as positive control.

### Apoptosis measurement

Inflammasome activation induces the production of pro-inflammatory cytokines leading to the apoptosis. To see the effect of upregulated inflammasome forming genes and upregulated pro-inflammatory genes expression in HIV infected macrophages exposed to the cocaine, we have measured the apoptosis in these cells. Monocyte Derived Macrophages were infected with HIV for 10 days in combination with cocaine. At the termination of 10 days infection and/or cocaine treatment, cells were washed twice with cold PBS and then resuspended in 1X binding buffer at a concentration of 1 × 10^6^ cells/ml. 100 μl from this is added to a 5 ml FACS tubes, followed by incubation with 5 μl each of Annexin V and 7-aminoactinomycin D (7-AAD) (BD Biosciences, FITC Annexin V Apoptosis Detection Kit I, Cat # 556547) for 15 minutes at RT in the dark. After incubation, 400 μl of 1X binding buffer is added to each tube, mixed gently and analyzed by FACScalibur within 1 hr. The untreated cells, which served as control, is used for defining the basal level of apoptotic and dead cells. The percentage of cells that have been induced to undergo apoptosis is then determined by subtracting the percentage of apoptotic cells in the untreated population from percentage of apoptotic cells in the treated population. Results were analyzed by using the Flowjo software and obtained test samples results were manually compensated based on single stain control.

### Data Analysis

In the expression studies, a gene was considered differentially regulated if the difference was ≥2 fold in comparison with the control. Experiments were performed in three independent biological experiments with triplicates and the values obtained were averaged. Relative density of the detected protein bands in western blots were measured by using the Image J software. All the results were expressed as mean ± s.e.m. Statistical analysis of two groups was performed by Student’s t test, while more than two groups were analyzed using one way ANOVA followed by Bonferroni’s multiple comparison test. Differences were considered significant at p ≤ 0.05. Data analysis was performed with the Statistical Program, GraphPad Prism software (La Jolla, CA).

### Ethics Statement

Leukopacks (buffy coats) were commercially obtained from the community blood bank (One Blood, Miami, FL, USA), for which ethics committee approval is not required.

## Results and Discussion

### MTT assay and cytotoxicity

**Figure 2:**
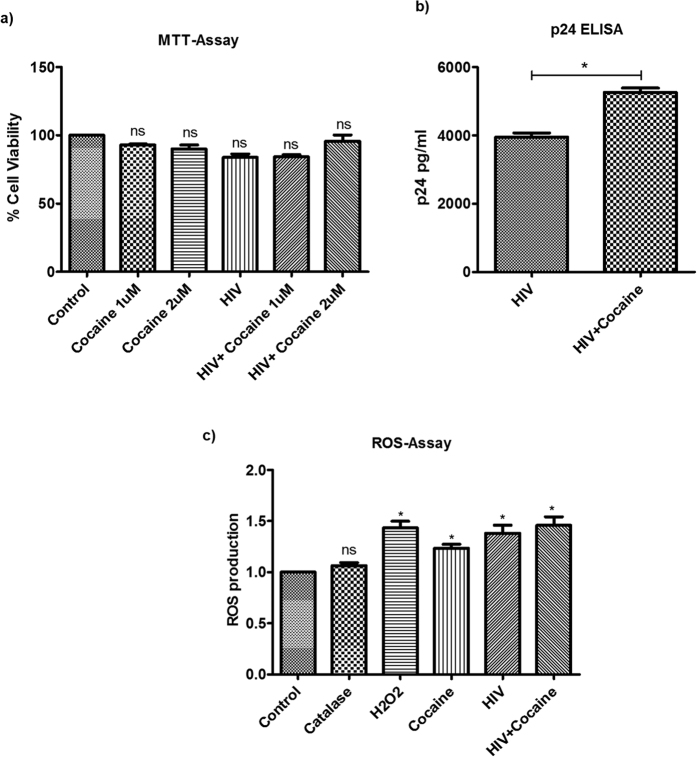
(**a**) MTT Assay. Monocyte derived macrophages were infected with HIV and/or treated with cocaine for 10 days and the cell viability was measured using the MTT assay. We have found no significant cytotoxicity in the macrophages at the concentration of cocaine or HIV used in the study. (NS-Not Significant). (**b**) Increased HIV infectivity of MDMs in the presence of cocaine. Monocyte derived macrophages were infected with HIV in the presence/absence of cocaine for 10 days and the HIV infectivity was measured by measuring the p24 antigen production in the culture supernatant using the p24 antigen ELISA Kit. We have found significantly increased HIV infectivity in macrophages exposed to cocaine than the HIV infected cells alone. (*p ≤ 0.05). (**c**) ROS Assay. Monocyte derived macrophages were infected with HIV and/or treated with cocaine for 10 days and oxidative stress was analyzed by ROS assay. We have found significant ROS production in cocaine alone treated, HIV only infected and HIV plus cocaine treated macrophages. (*p ≤ 0.05; NS-Not Significant).

Macrophages were infected with HIV in combination with different concentrations of cocaine (1 μM and 2 μM) for 10 days. Cytotoxicity was measured by MTT assay and we have not seen any significant cytotoxicity in any of the test group (cocaine/HIV alone and in HIV plus cocaine combined treatment) macrophages when compared with the control cells (Fig. 2a). Based on these cytotoxicity report, we have used 1 uM concentration of cocaine in our further experiments.

### Increased macrophages HIV-1 infectivity in the presence of cocaine

Macrophages were infected with HIV-1 for 10 days in the presence or absence of cocaine. HIV infectivity was measured by quantifying the amount of p24 antigen released into the culture supernatant. We have observed significantly increased HIV infection in the presence of cocaine (p < 0.05) when compared to the HIV alone-infected cells (Fig. 2b). Our results are in agreement with the previous reports showing the increased replication of HIV in the monocyte derived macrophages exposed to cocaine^17^. Reports suggest that immunomodulatory functions of cocaine may positively contribute to the increased HIV-1 infection and replication^18^,^19^. Also, cocaine has been reported to increase the expression of co-receptors [C-X-C chemokine receptor type 4 (CXCR4) and C-C chemokine receptor type 5 (CCR5)] for HIV in mononuclear cells^20^. In this study, we have analyzed the expression of different HIV restriction factors and their possible role in the increased HIV infectivity in MDMs exposed to cocaine. Further, we have analyzed the expression of different inflammasome participating genes and their regulating and down-stream signaling genes to explore the mechanisms of HIV pathogenesis in cocaine abusers.

### HIV restriction factors expression in macrophages infected with HIV in the presence of cocaine

Mammalian cells express a number of diverse, dominantly acting proteins that are widely expressed and function in a cell-autonomous manner to suppress HIV replication. These include BST2, TRIM5α, and APOBEC3G. To see the effect of cocaine on the expression of these HIV restriction factors in HIV infected macrophages, HIV infected macrophages were treated with cocaine for 10 days and observed for the expression of these restriction factors.

**Figure 3:**
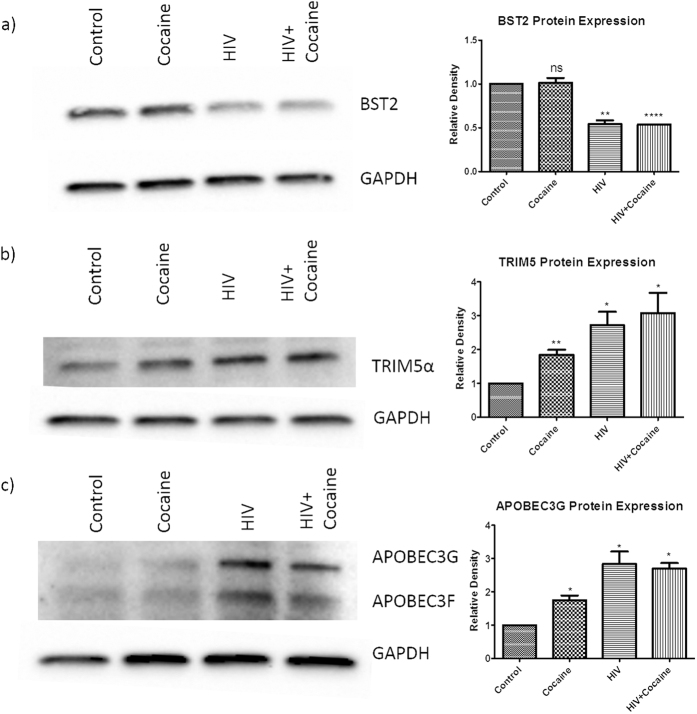
Expression of HIV restriction factors in HIV infected MDMs in the presence of cocaine. Monocyte derived macrophages were infected with HIV and/or treated with cocaine for 10 days and the protein expression of different HIV restriction factors (BST2, TRIM5α and APOBEC3G) was analyzed by western blot assay. We have found significant downregulation of BST2 restriction factor in cocaine alone treated, HIV only infected and HIV plus cocaine treated macrophages (**a**). We have also observed significant upregulation of TRIM5α (**b**) and APOBEC3G (**c**) protein expression in cocaine alone treated, HIV only infected and HIV plus cocaine treated macrophages. Figure 3 is the representative figure of three independent biological experiments. Relative density of the detected protein band was measured by using the Image J software and the values obtained were averaged. (*p ≤ 0.05; **p ≤ 0.01; ***p ≤ 0.001; NS-Not Significant).

BST2 or Tetherin is an interferon (IFN)-inducible transmembrane and glycosylphosphatidylinositol (GPI)-anchored protein that restricts the release of HIV from infected cells. Although BST2 can inhibit the release of free virion and helps in cell-free infectivity, restriction of the cell-to-cell spread of virus is less effective^21^,^22^. The HIV-1 protein Vpu antagonizes the tetherin by a protein-protein interaction and this interaction serves to remove BST-2 from the plasma membrane^23^,^24^. In this study, we have found significant down-regulation of BST2 protein in both HIV infected or cocaine alone treated and in combined treatment when compared with the control cells. We did not observe significant downregulation of BST2 in combined treatment when compared to the cocaine or HIV alone treated cells (Fig. 3a). These results indicate that down-regulation of Tetherin in HIV infected and HIV plus cocaine treated MDMs will help in further release of virions from the infected cells and facilitates the infection of the healthy neighboring MDMs.

TRIM5α was first identified as the protein responsible for host restriction of HIV-1 in rhesus macaque cells. TRIM5α reported to inhibit HIV-1 infection by targeting the viral capsid following entry, and its association with viral capsid leads to the premature uncoating or degradation of the viral capsid^25^,^26^,^27^,^28^,^29^,^30^. We have observed significant upregulation of TRIM5α in cocaine/HIV alone treated and in combined treatment when compared to the control cells (Fig. 3b). Although we have seen up-regulation of TRIM5α expression in HIV plus cocaine treated cells than HIV alone infected cells, statistically it is not significant.

APOBEC3G protein severely restricts the replication of HIV by extensively deaminating cytosine residues in the viral genome during reverse transcription. This process introduces mutations (cytosine-to-uracil) in the –ve strand viral DNA, leading to either the destabilization of reverse transcripts or the production of defective viral proteins^31^,^32^,^33^. Although APOBEC3G is a potent antiviral molecule, HIV-1 can counteract with its accessory protein, viral infectivity factor (Vif). In infected cells, Vif forms an ubiquitin ligase complex with Cullin5 (CUL5), Elongin B/C (ELOB/C) and Core binding factor β (CBFβ) that ubiquitinates and degrades APOBEC3G^34^,^35^,^36^. In this study, up-regulated TRIM5α (Fig. 3b) and APOBEC3G in HIV or HIV plus cocaine treated cells (Fig. 3c) may indicate the cell combat mechanism against the HIV to restrict the increased infection. TRIM5α mRNA expression levels were reported to be lower in the PBMCs of HIV-1-infected subjects than in those of uninfected subjects and seroconverters reported to have lower pre-infection levels of TRIM5alpha than did non-seroconverters^37^. Along with these three restriction factors, we have also analyzed the expression of SAM domain and HD domain-containing protein 1 (SAMHD1) in these HIV infected macrophages in combination with cocaine. But we did not see the detectable levels of SAMHD1 expression in these primary macrophages in our experiments.

### Increased ROS production in HIV and/or cocaine treated cells

ROS are a group of highly reactive free radicals, produced mainly by phagocytic cells such as neutrophils and macrophages^38^. An increased oxidative stress condition has been repeatedly described in chronically HIV-1-infected patients, based on: elevated extracellular and intracellular ROS levels and ROS production is one of the indirect-activators of inflammasomes formation. Therefore, to see the effect of cocaine on HIV induced ROS production and thereby inflammasome formation, we have treated the HIV infected macrophages with cocaine and observed for oxidative stress by measuring the ROS production. We have observed significantly increased ROS production in HIV infected and/or cocaine treated cells when compared to the control cells. But we did not find additive or synergistically increased ROS production in HIV+ cocaine treated cells when compared to the HIV/cocaine alone treated cells (Fig. 2c). These results indicate that increased ROS production may be one of the factors responsible for the activation of inflammasomes in HIV and/or cocaine treated macrophages.

### Expression of inflammasome forming genes, regulatory genes and down-stream signaling genes in macrophages infected with HIV and/or treated with cocaine

Table 2 shows the list of dysregulated inflammasomes, NOD-like receptors, pro-inflammatory caspase genes and negative regulating and down-stream regulating genes in HIV and/or cocaine treated macrophages.

Pro-apoptotic gene, caspase 5 (CASP5) was significantly (≥2 fold) down-regulated in cocaine treated macrophages, and it belongs to the IPAF (NLRC4) inflammasomes family. While cocaine reported to induce the apoptosis in macrophages^39^, the effect of Caspase 5 down-regulation in cocaine treated macrophages need to be further investigated. In HIV infected macrophages, AIM2 gene expression was significantly up-regulated. The inflammasome negative regulatory genes CD40LG (TNFSF5) and PYDC1 (POP1) were significantly down-regulated in HIV infected macrophages, indicating the possible AIM2 mediated inflammasome formation in the HIV infected macrophages. The AIM2 recognizes cytosolic dsDNA inflammasome, can be activated by viral DNA to trigger caspase-1. AIM2 upregulation was reported in chronic hepatitis B patients and its expression is correlated with HBV-associated inflammatory activity^40^. In HIV infected macrophages treated with cocaine, NLRP3 and AIM2 genes were significantly up-regulated, whereas inflammasome negative regulatory genes CD40LG and PYDC1 genes were significantly down-regulated. Basal expression of pro-form of IL-1β as well as the NLRP3 protein is barely detectable. In the presence of virus/bacteria/PAMPS, their transcription is initiated^41^. The NLRP3 inflammasome acts as an early mediator of inflammation by cleaving proforms of IL-1β and IL-18 and releasing the active forms. In this study, we have observed the significant upregulation of NLRP3 and the downstream inflammatory factor IL-1β in HIV infected cocaine exposed macrophages. These observations suggest the involvement of NLRP3 inflammasome in the onset and development of the neuroinflammation in cocaine abusing HIV patients. These results indicate the possible involvement of both NLRP3 and AIM2 mediated inflammasomes in case of HIV plus cocaine treated cells.

### Expression of NOD like receptors, regulatory gens and down-stream signaling genes in macrophages infected with HIV and/or treated with cocaine

In macrophages exposed to cocaine alone, CCL2/Monocyte chemotactic protein 1 (MCP-1) (−2.1 fold), IL6 (−2.51 fold) were the two downstream signaling genes significantly down-regulated when compared to the control cells. In case of HIV infected cells, NOD2 (+2.79 fold) and the downstream signaling gene CCL7 (+11 fold) were significantly up-regulated. In case of HIV infected cells exposed to cocaine, NOD2 (+2.77 fold) and NLRP3 (+2.07 fold) were significantly up-regulated and the downstream signaling genes CCL2 (+2.01 fold), CCL7 (+32 fold), IL6 (+9 fold) were also significantly upregulated.

In cocaine alone treated cells, we have observed down-regulation of interleukin 6 (IL-6) which acts as both pro-inflammatory cytokine as well as an anti-inflammatory myokine. In particular, IL-6 is involved in differentiation of B cells, antibody production, activation of T cells, hematopoiesis, pyrogenesis, corticotropin-releasing factor (CRF) stimulation, induction of acute phase proteins in the liver, as well as other neuroendocrine changes^42^,^43^. IL-6 also has significant indirect anti-inflammatory properties^44^. Cocaine-induced suppression of proinflammatory IL-6 may mediate impaired host defenses to infections^45^. In case of HIV infected macrophages incubated with cocaine, we have observed 9 fold upregulation of IL-6. Few earlier reports also have reported the elevated IL-6 production in *in vitro* infected monocytic lineage^46^ as well as in HIV infected patient plasma^47^ cerebrospinal fluid samples^48^. Also, IL-6 alone has been reported to stimulate the HIV-1 replication in macrophages^49^.

NOD2 [caspase recruitment domain-containing protein 15 (CARD15)] is an intracellular PRR and macrophage specific protein containing two CARD domains, a large nucleotide binding domain and leucine-rich repeats^50^. Activation of NOD2 results in initiation of both innate and acquired immune responses and the enzymatic cleavage of pro-IL-1β, which releases the biologically active form of IL-1β^51^,^52^. In case of HIV alone infected macrophages, we have observed significant upregulation of NOD2 expression than the control cells. Where as in case of HIV infected plus cocaine treated cells, we have observed upregulation of both NOD2 and IL-1β expression, indicating their possible role in apoptosis in these cells.

Monocyte chemoattractant proteins (MCPs), which belong to the beta chemokine family, and especially CCL2 (MCP1) and its receptor CCR2 have been implicated in the neurological disorders^53^. Monocyte/macrophages are found to be the major source of CCL2^54^ and regulates the migration and infiltration of monocytes, memory T lymphocytes, natural killer (NK) cells, dendritic cells into foci of active inflammation^55^,^56^. These β-chemokines are well known to induce the neuroinflammation^57^. In this study, in HIV infected MDMs exposed to cocaine (but not in HIV only infected cells), we have observed significant up-regulation of CCL2 expression than the control cells. Surprisingly in cocaine alone treated cells, we have seen significant down-regulation of CCL2 expression. These results indicate the significant CCL2 mediated inflammatory response in the HIV infected cocaine abusers.

Monocyte Chemoattractant Protein 3 (MCP-3), also called CCL7, is produced by macrophages and some tumor cell lines. CCL7 is regarded as one of the most pluripotent chemokines, as it can bind and signal via multiple CC chemokine receptors, including CCR1, CCR2 and CCR3^58^. CCL2 and CCL7 are predominantly associated with the egress of monocytes out of the bone marrow in response to proinflammatory stimuli^59^. In various disorders associated with increased inflammatory infiltrates like demyelinating diseases, simian virus induced acquired immune deficiency syndrome encephalitis, lymphocytic choriomeningitis, and MCAO (middle cerebral artery occlusion), upregulation of CCL7 was reported^60^. In this study we have observed the highly upregulation of CCL7 in HIV alone (11 fold) when compared to the control cells and synergistic upregulation in HIV plus cocaine treated cells (32 fold). Therefore our results show that upregulated CCL7 in HIV alone and HIV plus cocaine treated macrophages may be highly responsible in inducing the inflammatory response in the brain.

### Pro-Inflammatory Caspases in macrophages infected with HIV and/or treated with cocaine

Little is known about caspase-5, the mechanisms involved in its activation and physiological functions. CASP-5 is a part of the NLRP1 inflammasome complex^8^ and reported its role in induction of apoptosis^61^,^62^. Out of the two pro-inflammatory caspases we have analyzed we have only found significant downregulation of CASP5 (−3 fold) in cocaine treated macrophages as discussed earlier. In both HIV infected and HIV plus cocaine treated cells, we have observed upregulation (<2 fold) of both CASP1 and CASP5 genes. Processing of pro- IL-1β into the mature form of IL-1β is mediated by the enzyme CASP1 which is a part of the NLRP1, NLRP3, IPAF and AIM2 inflammasome complexes. These results indicate that upregulation of CASP1 and CASP5 genes in HIV or HIV plus cocaine treated macrophages induce the activation of the IL-1β that leads to the inflammatory response in these conditions.

### Apoptosis measurement in HIV infected macrophages in the presence of cocaine

**Figure 4:**
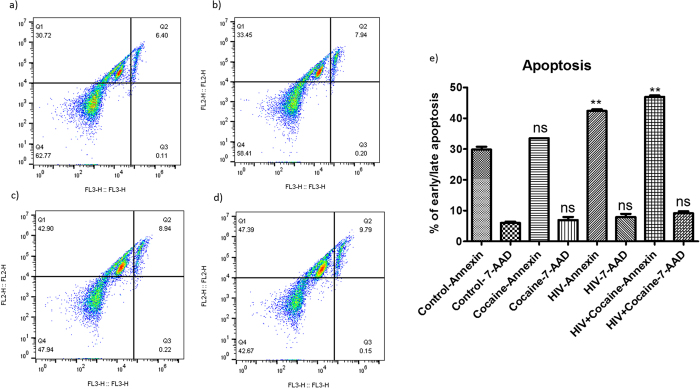
Apoptosis in HIV infected macrophages grown in the presence/absence of cocaine. Monocyte derived macrophages were infected with HIV in the presence/absence of cocaine for 10 days and apoptosis was measured by using FITC Annexin V Apoptosis Detection Kit I and the results were analyzed by Flow-cytometer. We have observed significant early apoptosis in HIV only infected macrophages (**c**) as well as in HIV infected macrophages grown in the presence of cocaine (**d**) when compared to the control cells (**a**). We did not see significant apoptosis in cocaine only exposed cells (**b**) when compared to the control cells. Although there is an increase but there is no statistically significantly increased early apoptosis in HIV infected cells grown in the presence of cocaine (**d**) in comparison with the HIV alone infected cells (**c**). No significant late apoptosis was observed in any of the test group cells when compared to the control cells (**e**). Figure 4 is the representative figure of three independent biological experiments and the values obtained were averaged. (**p ≤ 0.01; NS-Not Significant).

To see the effect of changes in inflammasomes and their regulated genes in HIV infected macrophages grown in the presence of cocaine, we have analyzed the apoptosis levels in these cells. We have observed significant early apoptosis in HIV infected and in HIV infected cells in the presence of cocaine when compared to the control cells. We did not observe significant early apoptosis in cocaine alone treated macrophages. There is no significantly increased early apoptosis levels in HIV plus cocaine treated cells when compared with the HIV alone infected cells. We did not see any significant change in the percentage of cells in late apoptosis in any of the test group when compared with the control cells (Fig. 4). HIV infected macrophages has been reported to induce apoptosis in bystander cells (CD4+ and CD8+ T cells) by releasing soluble cytotoxic factors^63^,^64^. Cocaine has been reported to induce the apoptosis in HIV infected macrophages by increasing the secretion of cathepsin B^39^. Role of production of different inflammasomes in HIV infected macrophages in the presence of cocaine on apoptosis induction needs to be further investigated.

## Conclusions

**Figure 5:**
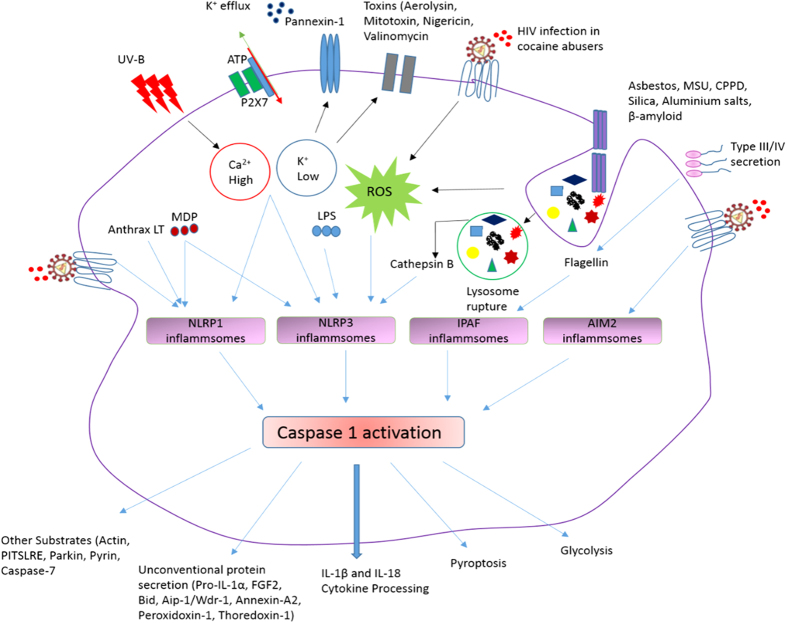
Inflammasome signaling cascade. This schematic representation is showing the cascade of different types of activators and inflammasome complexes and their mechanism of action in inducing inflammatory response and cell death. This figure shows that HIV infection and/or cocaine exposure induce the expression of NLRP1, NLRP3 (by ROS production), and AIM2 inflammasome genes which further activates the production of pro-inflammatory cytokines leading to the apoptosis of the infected cells and/or surrounding cell population. This figure also shows other activators of NLRP1 (K^+^ efflux, Ca^2+^ influx), NLRP3 (K^+^ efflux, cathepsin B, ROS production), IPAF (flagellin from certain gram negative bacteria), AIM2 (cytosolic bacterial, viral and host dsDNA). Courtesy of AdipoGen Life Sciences (www.adipogen.com).

Macrophages are initiators of an inflammatory cascade in HIV infection, which ultimately results in neuronal damage and dysfunction^65^,^66^; and increased neuroinflammation has been reported in HIV-infected cocaine abusers^67^. Therefore investigating the mechanisms of HIV infection and inflammatory responses in macrophages infected with HIV in combination with cocaine will help us in understanding the neuroinflammatory responses in HIV infected cocaine abusers. Our results concur the information on the increased HIV-1 infection levels in macrophages in the presence of cocaine. Further we have investigated the expression of different HIV restriction factors in HIV infected macrophages treated with cocaine. We have observed significant down-regulation of BST2 protein in HIV and HIV plus cocaine treated cells indicating one of the possible mechanism for the increased HIV infection in cocaine abusers. In HIV infected macrophages treated with cocaine, we have observed significant up-regulation of AIM2, NLRP3 that participate in the formation of inflammasome complexes which further induce the caspase-1 pathway and activation of pro-form of IL-1β to active IL-1β which leads to the inflammatory response and cell death. Figure 5 shows the schematic representation of effect of HIV infection and other activators on inflammasome formation and their cascade that leads to the inflammatory responses and cell death in macrophages. In HIV infected macrophages in the presence of cocaine, CCL7 and IL-6 are the highly up-regulated genes and persistent high IL-6 production has been implicated in the increased HIV replication, development of various autoimmune, chronic inflammatory diseases^49^,^68^,^69^,^70^. Down-regulation of inflammasome negative regulatory genes CD40LG and PYDC1 in HIV plus cocaine treated cells may also facilitates the formation of NLRP3 and AIM2 mediated inflammatory response. Further, we have found that in case of HIV infected macrophages exposed to cocaine, increased ROS production and subsequent upregulated IL-1β transcription may serves as an activators for the formation of NLRP3 and AIM2 mediated inflammasomes that results in Caspase 1 activation which may be one of the mechanisms for severe neuroinflmmatory response and apoptosis of infected cells in HIV infected cocaine abusers. Further genetic and pharmacological approaches are necessary to investigate the inflammsome activators in HIV infected cocaine abusers for better understanding the inflammatory/neuroinflammatory response observed in these patients.

## Additional Information

**How to cite this article**: Atluri, V. S. R. *et al*. Effect of Cocaine on HIV Infection and Inflammasome Gene Expression Profile in HIV Infected Macrophages. *Sci. Rep.* **6**, 27864; doi: 10.1038/srep27864 (2016).

## Figures and Tables

**Figure 1:**
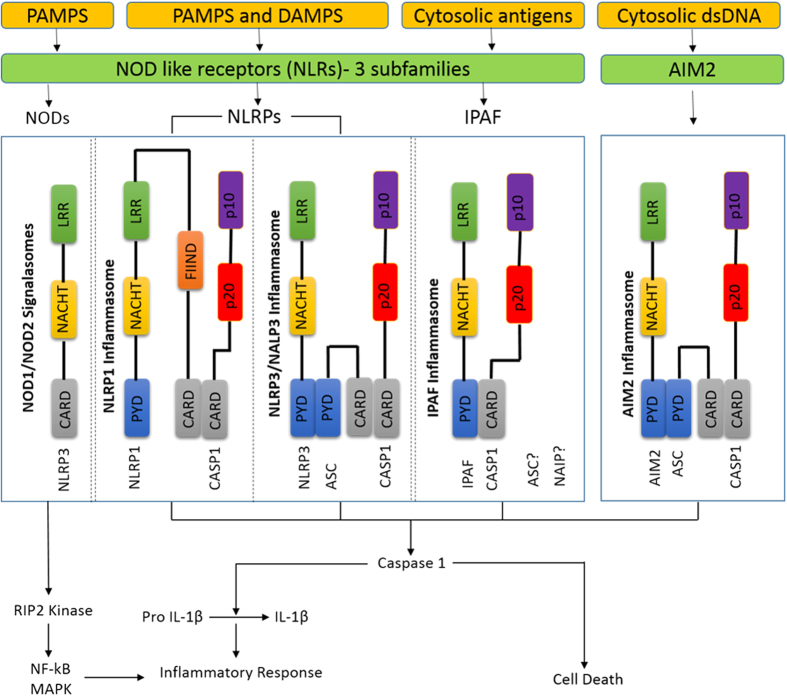
Danger signals or bacterial compounds as activators of inflammasomes and NODs. Schematic representation is showing different types and the components of inflammasomes and NOD like receptors. Courtesy of AdipoGen Life Sciences (www.adipogen.com).

**Table 1:** List of Inflammasomes, NOD-like receptors, Pro-inflammatory caspase genes and negative regulating and down-stream regulating genes.

Group	Genes
1. Inflammasomes	a) NLRP1: CASP1 (ICE), CASP5, NLRP1.
	b) NLRP3: CASP1 (ICE), NLRP3, PYCARD (ASC).
	c) IPAF: CASP1, NAIP, NLRC4 (IPAF), PYCARD (ASC).
	d) AIM2: AIM2, CASP1 (ICE), PYCARD (ASC).
	e) Negative Regulation: BCL2, BCL2L1 (BCL-X), CARD18 (ICEBERG), CD40LG (TNFSF5), CTSB, HSP90AA1, HSP90AB1, HSP90B1 (TRA1), MEFV, PSTPIP1, PYDC1 (POP1), SUGT1, TNF, TNFSF11, TNFSF14, TNFSF4 (OX40L).
	f) Downstream Signaling: IFNG, IL12A, IL12B, IL18, IL1B, IL33, IRAK1, IRF1, MYD88, P2RX7, PANX1, PTGS2 (COX2), MOK, RIPK2, TIRAP, TXNIP.
2. NOD-Like Receptors	a) Receptors: CIITA, NAIP (BIRC1), NLRC4 (IPAF), NLRC5, NLRP1, NLRP12, NLRP3, NLRP4, NLRP5, NLRP6, NLRP9, NLRX1, NOD1 (CARD4), NOD2.
	b) Downstream Signaling: BIRC2 (c-IAP2), BIRC3 (c-IAP1), CARD6, CASP8 (FLICE), CCL2 (MCP-1), CCL5 (RANTES), CCL7 (MCP-3), CFLAR (CASPER), CHUK (IKKa), CXCL1, CXCL2, FADD, IFNB1, IKBKB, IKBKG, IL6, IRF1, IRF2, MAP3K7 (TAK1), MAPK1 (ERK2), MAPK11, MAPK12, MAPK13, MAPK3 (ERK1), MAPK8 (JNK1), MAPK9 (JNK2), NFKB1, NFKBIA (I?Ba/MAD3), NFKBIB (TRIP9), PEA15, RELA, RIPK2, SUGT1, TAB1 (MAP3K7IP1), TAB2 (MAP3K7IP2), TNF, TRAF6, XIAP.
3. Pro-Inflammatory Caspases	CASP1 (ICE), CASP5.

**Table 2:** List of significantly dysregulated Inflammasomes, NOD-like receptors, Pro-inflammatory caspase genes and negative regulating and down-stream regulating genes expression fold change in HIV infected and/or cocaine treated monocyte derived macrophages.

Genes	Cocaine	HIV	HIV+ Cocaine
AIM2^1d^	−1.76	2.28	2.48
CASP5^1a,3^	−3.09	1.09	1.64
CCL2^2b^	−2.10	1.73	2.01
CCL7^2b^	−1.06	11.07	32.00
CD40LG^1e^	−1.41	−3.01	−2.71
IL12A^1f^	1.71	−2.59	−1.71
IL1B^1f^	−1.31	1.33	4.26
IL18	−1.12	−1.68	−1.73
IL6^2b^	−2.51	1.12	9.06
MEFV^1e^	−1.05	1.83	3.36
NLRP3^1b,2a^	1.28	1.31	2.07
NOD2^2a^	1.02	2.79	2.77
PTGS2^1f^	1.08	−1.87	2.00
PYDC1^1e^	1.71	−2.02	−2.48

## References

[b1] Wu D. T. . Berman. Mechanisms of leukocyte trafficking into the CNS. Journal of NeuroVirology 6 (Suppl. 1), S82–S85 (2000). 10871769

[b2] Nath A., Conant K., Chen P., Scott C. & Major E. O. Transient exposure to HIV-1 Tat protein results in cytokine production in macrophages and astrocytes. A hit and run phenomenon. J Biol Chem 274, 17098–17102, 10.1074/jbc.274.24.17098 (1999). 10358063

[b3] Gannon P., Khan M. Z. & Kolson D. L. Current understanding of HIV-associated neurocognitive disorders pathogenesis. Curr Opin Neurol 24, 275–283, 10.1097/WCO.0b013e32834695fb (2011). 21467932 PMC3683661

[b4] Buch S. . Cocaine and HIV-1 interplay in CNS: cellular and molecular mechanisms. Curr HIV Res 10, 425–428, 10.2174/157016212802138823 (2012). 22591366 PMC3824955

[b5] Kawai T. & Akira S. TLR signaling. Seminars in immunology 19, 24–32, 10.1016/j.smim.2006.12.004 (2007). 17275323

[b6] Kufer T. A. & Sansonetti P. J. Sensing of bacteria: NOD a lonely job. Current opinion in microbiology 10, 62–69, 10.1016/j.mib.2006.11.003 (2007). 17161646

[b7] Martinon F. & Tschopp J. Inflammatory caspases and inflammasomes: master switches of inflammation. Cell death and differentiation 14, 10–22, 10.1038/sj.cdd.4402038 (2007). 16977329

[b8] Martinon F., Burns K. & Tschopp J. The inflammasome: a molecular platform triggering activation of inflammatory caspases and processing of proIL-beta. Molecular cell 10, 417–426, 10.1016/S1097-2765(02)00599-3 (2002). 12191486

[b9] Schroder K. & Tschopp J. The Inflammasomes. Cell 140, 821–832, 10.1016/j.cell.2010.01.040 (2010). 20303873

[b10] Bergsbaken T., Fink S. L. & Cookson B. T. Pyroptosis: host cell death and inflammation. Nat Rev Microbiol 7, 99–109, 10.1038/nrmicro2070 (2009). 19148178 PMC2910423

[b11] Zybarth G., Reiling N., Schmidtmayerova H., Sherry B. & Bukrinsky M. Activation-Induced Resistance of Human Macrophages to HIV-1 Infection *In Vitro*. The Journal of Immunology 162, 400–406 (1999). 9886413

[b12] Nasr N. . HIV-1 infection of human macrophages directly induces viperin which inhibits viral production. 120, 778–88, 10.1182/blood-2012-01-407395 (2012). 22677126

[b13] Kurapati K. R. V., Atluri V. S. R., Samikkannu T. & Nair M. P. N. Ashwagandha (Withania somnifera) Reverses β-Amyloid1-42 Induced Toxicity in Human Neuronal Cells: Implications in HIV-Associated Neurocognitive Disorders (HAND). PLoS One 8, e77624, 10.1371/journal.pone.0077624 (2013). 24147038 PMC3797707

[b14] Atluri V. S., Kanthikeel S. P., Reddy P. V., Yndart A. & Nair M. P. Human synaptic plasticity gene expression profile and dendritic spine density changes in HIV-infected human CNS cells: role in HIV-associated neurocognitive disorders (HAND). PLoS One 8, e61399, 10.1371/journal.pone.0061399 (2013). 23620748 PMC3631205

[b15] Yndart A. . Bath salts alter synaptic plasticity gene expression in neurons. Journal of NeuroImmune Pharmacology 9, 62, 10.1007/s11481-014-9535-3 (2014).

[b16] Agudelo M. . Effects of Alcohol on Histone Deacetylase 2 (HDAC2) and the Neuroprotective Role of Trichostatin A (TSA). Alcoholism, clinical and experimental research 35, 1550–1556, 10.1111/j.1530-0277.2011.01492.x (2011). PMC3128172 21447001

[b17] Dhillon N. K. . Cocaine-mediated enhancement of virus replication in macrophages: implications for human immunodeficiency virus-associated dementia. J Neurovirol 13, 483–495, 10.1080/13550280701528684 (2007). 18097880

[b18] Nair M. P. . Cocaine differentially modulates chemokine production by mononuclear cells from normal donors and human immunodeficiency virus type 1-infected patients. Clin Diagn Lab Immunol 7, 96–100 (2000). 10618285 10.1128/cdli.7.1.96-100.2000 PMC95830

[b19] Roth M. D., Whittaker K. M., Choi R., Tashkin D. P. & Baldwin G. C. Cocaine and sigma-1 receptors modulate HIV infection, chemokine receptors, and the HPA axis in the huPBL-SCID model. J Leukoc Biol 78, 1198–1203, 10.1189/jlb.0405219 (2005). 16204638

[b20] Nair M. P. . Effect of cocaine on chemokine and CCR-5 gene expression by mononuclear cells from normal donors and HIV-1 infected patients. Adv Exp Med Biol 493, 235–240, 10.1007/0-306-47611-8_28 (2001). 11727771

[b21] Casartelli N. . Tetherin restricts productive HIV-1 cell-to-cell transmission. PLoS Pathog 6, e1000955, 10.1371/journal.ppat.1000955 (2010). 20585562 PMC2887479

[b22] Jolly C., Booth N. J. & Neil S. J. Cell-cell spread of human immunodeficiency virus type 1 overcomes tetherin/BST-2-mediated restriction in T cells. J Virol 84, 12185–12199, 10.1128/JVI.01447-10 (2010). 20861257 PMC2976402

[b23] Neil S. J., Zang T. & Bieniasz P. D. Tetherin inhibits retrovirus release and is antagonized by HIV-1 Vpu. Nature 451, 425–430, 10.1038/nature06553 (2008). 18200009

[b24] Van Damme N. . The interferon-induced protein BST-2 restricts HIV-1 release and is downregulated from the cell surface by the viral Vpu protein. Cell Host Microbe 3, 245–252, 10.1016/j.chom.2008.03.001 (2008). 18342597 PMC2474773

[b25] Besnier C., Takeuchi Y. & Towers G. Restriction of lentivirus in monkeys. Proc Natl Acad Sci USA 99, 11920–11925, 10.1073/pnas.172384599 (2002). 12154231 PMC129369

[b26] Berthoux L., Sebastian S., Sokolskaja E. & Luban J. Lv1 inhibition of human immunodeficiency virus type 1 is counteracted by factors that stimulate synthesis or nuclear translocation of viral cDNA. J Virol 78, 11739–11750, 10.1128/JVI.78.21.11739-11750.2004 (2004). 15479815 PMC523245

[b27] Besnier C. . Characterization of murine leukemia virus restriction in mammals. J Virol 77, 13403–13406, 10.1128/JVI.77.24.13403-13406.2003 (2003). 14645596 PMC296090

[b28] Munk C., Brandt S. M., Lucero G. & Landau N. R. A dominant block to HIV-1 replication at reverse transcription in simian cells. Proc Natl Acad Sci USA 99, 13843–13848, 10.1073/pnas.212400099 (2002). 12368468 PMC129785

[b29] Stremlau M. . The cytoplasmic body component TRIM5alpha restricts HIV-1 infection in Old World monkeys. Nature 427, 848–853, 10.1038/nature02343 (2004). 14985764

[b30] Stremlau M. . Specific recognition and accelerated uncoating of retroviral capsids by the TRIM5alpha restriction factor. Proc Natl Acad Sci USA 103, 5514–5519, 10.1073/pnas.0509996103 (2006). 16540544 PMC1459386

[b31] Harris R. S. . DNA deamination mediates innate immunity to retroviral infection. Cell 113, 803–809, 10.1016/S0092-8674(03)00423-9 (2003). 12809610

[b32] Mangeat B. . Broad antiretroviral defence by human APOBEC3G through lethal editing of nascent reverse transcripts. Nature 424, 99–103, 10.1038/nature01709 (2003). 12808466

[b33] Zhang H. . The cytidine deaminase CEM15 induces hypermutation in newly synthesized HIV-1 DNA. Nature 424, 94–98, 10.1038/nature01707 (2003). 12808465 PMC1350966

[b34] Yu X. . Induction of APOBEC3G ubiquitination and degradation by an HIV-1 Vif-Cul5-SCF complex. Science 302, 1056–1060, 10.1126/science.1089591 (2003). 14564014

[b35] Zhang W., Du J., Evans S. L., Yu Y. & Yu X. F. T-cell differentiation factor CBF-beta regulates HIV-1 Vif-mediated evasion of host restriction. Nature 481, 376–379, 10.1038/nature10718 (2012). 22190036

[b36] Jager S. . Vif hijacks CBF-beta to degrade APOBEC3G and promote HIV-1 infection. Nature 481, 371–375, 10.1038/nature10693 (2012). 22190037 PMC3310910

[b37] Sewram S. . Human TRIM5alpha expression levels and reduced susceptibility to HIV-1 infection. J Infect Dis 199, 1657–1663, 10.1086/598861 (2009). 19388851 PMC2725358

[b38] Lam G. Y., Huang J. & Brumell J. H. The many roles of NOX2 NADPH oxidase-derived ROS in immunity. Semin Immunopathol 32, 415–430, 10.1007/s00281-010-0221-0 (2010). 20803017

[b39] Zenon F., Segarra A. C., Gonzalez M. & Melendez L. M. Cocaine potentiates cathepsin B secretion and neuronal apoptosis from HIV-infected macrophages. J Neuroimmune Pharmacol 9, 703–715, 10.1007/s11481-014-9563-z (2014). 25209871 PMC4209444

[b40] Han Y. . Expression of AIM2 is correlated with increased inflammation in chronic hepatitis B patients. Virology Journal 12, 129, 10.1186/s12985-015-0360-y (2015). 26290184 PMC4545983

[b41] Bauernfeind F. . Reactive oxygen species inhibitors block priming, but not activation of the NLRP3 inflammasome. Journal of immunology (Baltimore, Md.: 1950) 187, 613–617, 10.4049/jimmunol.1100613 (2011). PMC3131480 21677136

[b42] Papanicolaou D. A., Wilder R. L., Manolagas S. C. & Chrousos G. P. The pathophysiologic roles of interleukin-6 in human disease. Ann Intern Med 128, 127–137, 10.7326/0003-4819-128-2-199801150-00009 (1998). 9441573

[b43] Hirano T., Akira S., Taga T. & Kishimoto T. Biological and clinical aspects of interleukin 6. Immunol Today 11, 443–449 (1990). 2127356 10.1016/0167-5699(90)90173-7

[b44] Lyson K. & McCann S. M. The effect of interleukin-6 on pituitary hormone release *in vivo* and *in vitro*. Neuroendocrinology 54, 262–266 (1991). 1658674 10.1159/000125884

[b45] Halpern J. H. . Diminished Interleukin-6 Response to Proinflammatory Challenge in Men and Women after Intravenous Cocaine Administration. The Journal of Clinical Endocrinology & Metabolism 88, 1188–1193, 10.1210/jc.2002-020804 (2003). 12629105

[b46] Nakajima K. . Induction of IL-6 (B cell stimulatory factor-2/IFN-beta 2) production by HIV. J Immunol 142, 531–536 (1989). 2783441

[b47] Breen E. C. . Infection with HIV is associated with elevated IL-6 levels and production. The Journal of Immunology 144, 480–484 (1990). 2295799

[b48] Gallo P. . Human immunodeficiency virus type 1 (HIV-1) infection of the central nervous system: an evaluation of cytokines in cerebrospinal fluid. Journal of Neuroimmunology 23, 109–116, 10.1016/0165-5728(89)90029-5 (1989). 2656753

[b49] Poli G. . Interleukin 6 induces human immunodeficiency virus expression in infected monocytic cells alone and in synergy with tumor necrosis factor alpha by transcriptional and post-transcriptional mechanisms. J Exp Med 172, 151–158 (1990). 2193094 10.1084/jem.172.1.151 PMC2188185

[b50] Ogura Y. . Nod2, a Nod1/Apaf-1 family member that is restricted to monocytes and activates NF-kappaB. J Biol Chem 276, 4812–4818, 10.1074/jbc.M008072200 (2001). 11087742

[b51] Hasegawa M. . A critical role of RICK/RIP2 polyubiquitination in Nod-induced NF-Î°B activation. The EMBO Journal 27, 373–383, 10.1038/sj.emboj.7601962 (2008). 18079694 PMC2234345

[b52] Magalhaes J. G. . Essential role of Rip2 in the modulation of innate and adaptive immunity triggered by Nod1 and Nod2 ligands. European Journal of Immunology 41, 1445–1455, 10.1002/eji.201040827 (2011). 21469090

[b53] Conductier G. g., Blondeau N., Guyon A., Nahon J.-L. & RovÃ¨re C. The role of monocyte chemoattractant protein MCP1/CCL2 in neuroinflammatory diseases. Journal of Neuroimmunology 224, 93–100, 10.1016/j.jneuroim.2010.05.010 (2010). 20681057

[b54] Yoshimura T., Robinson E. A., Tanaka S., Appella E. & Leonard E. J. Purification and amino acid analysis of two human monocyte chemoattractants produced by phytohemagglutinin-stimulated human blood mononuclear leukocytes. J Immunol 142, 1956–1962 (1989). 2921521

[b55] Carr M. W., Roth S. J., Luther E., Rose S. S. & Springer T. A. Monocyte chemoattractant protein 1 acts as a T-lymphocyte chemoattractant. Proc Natl Acad Sci USA 91, 3652–3656 (1994). 8170963 10.1073/pnas.91.9.3652 PMC43639

[b56] Xu L. L., Warren M. K., Rose W. L., Gong W. & Wang J. M. Human recombinant monocyte chemotactic protein and other C-C chemokines bind and induce directional migration of dendritic cells *in vitro*. J Leukoc Biol 60, 365–371 (1996). 8830793 10.1002/jlb.60.3.365

[b57] Chui R. & Dorovini-Zis K. Regulation of CCL2 and CCL3 expression in human brain endothelial cells by cytokines and lipopolysaccharide. J Neuroinflammation 7, 1, 10.1186/1742-2094-7-1 (2010). 20047691 PMC2819252

[b58] Brunner P. M. . CCL7 contributes to the TNF-alpha-dependent inflammation of lesional psoriatic skin. Experimental Dermatology 24, 522–528, 10.1111/exd.12709 (2015). 25828150

[b59] Tsou C. L. . Critical roles for CCR2 and MCP-3 in monocyte mobilization from bone marrow and recruitment to inflammatory sites. J Clin Invest 117, 902–909, 10.1172/JCI29919 (2007). 17364026 PMC1810572

[b60] Thompson W. L. & Eldik L. J. V. Inflammatory cytokines stimulate the chemokines CCL2/MCP-1 and CCL7/MCP-7 through NFÎ°B and MAPK dependent pathways in rat astrocytes. Brain research 1287, 47–57, 10.1016/j.brainres.2009.06.081 (2009). 19577550 PMC2725204

[b61] Munday N. A. . Molecular cloning and pro-apoptotic activity of ICErelII and ICErelIII, members of the ICE/CED-3 family of cysteine proteases. J Biol Chem 270, 15870–15876 (1995). 7797592 10.1074/jbc.270.26.15870

[b62] Faucheu C., Blanchet A. M., Collard-Dutilleul V., Lalanne J. L. & Diu-Hercend A. Identification of a cysteine protease closely related to interleukin-1 beta-converting enzyme. Eur J Biochem 236, 207–213 (1996). 8617266 10.1111/j.1432-1033.1996.t01-1-00207.x

[b63] Badley A. D. . Macrophage-dependent Apoptosis of CD4+ T Lymphocytes from HIV-infected Individuals Is Mediated by FasL and Tumor Necrosis Factor. The Journal of Experimental Medicine 185, 55–64, 10.1084/jem.185.1.55 (1997). 8996241 PMC2196110

[b64] Herbein G. . Apoptosis of CD8+ T cells is mediated by macrophages through interaction of HIV gp120 with chemokine receptor CXCR4. Nature 395, 189–194 (1998). 9744279 10.1038/26026

[b65] Kraft-Terry S. . Proteomic analyses of monocytes obtained from Hispanic women with HIV-associated dementia show depressed antioxidants. Proteomics. Clinical applications 4, 706–714, 10.1002/prca.201000010 (2010). 21137088 PMC3098323

[b66] Vazquez-Santiago F. J., Noel R. Jr., Porter J. & Rivera-Amill V. Glutamate metabolism and HIV-associated neurocognitive disorders. Journal of NeuroVirology 20, 315–331, 10.1007/s13365-014-0258-2 (2014). 24867611 PMC4098898

[b67] Buch S. . Cocaine and HIV-1 interplay: molecular mechanisms of action and addiction. J Neuroimmune Pharmacol 6, 503–515, 10.1007/s11481-011-9297-0 (2011). 21766222 PMC3208732

[b68] Kishimoto T. The biology of interleukin-6. Blood 74, 1–10 (1989). 2473791

[b69] Kishimoto T. Interleukin-6: from basic science to medicine–40 years in immunology. Annu Rev Immunol 23, 1–21, 10.1146/annurev.immunol.23.021704.115806 (2005). 15771564

[b70] Akira S., Taga T. & Kishimoto T. Interleukin-6 in biology and medicine. Adv Immunol 54, 1–78 (1993). 8379461 10.1016/s0065-2776(08)60532-5

